# Role of Inferior Turbinoplasty in Endoscopic Sinus Surgery: A Systematic Review

**DOI:** 10.7759/cureus.96988

**Published:** 2025-11-16

**Authors:** Suliman Ali, Avenie Mavadia, Alf Cuddeford, Zahra Mubaarak, Yadsan Devabalan, Chuanyu Gao, Remo Accorona, Haissan Iftikhar

**Affiliations:** 1 Otolaryngology, Northampton General Hospital, Northampton, GBR; 2 Otolaryngology, Azienda Socio Sanitaria Territoriale (ASST) Grande Ospedale Metropolitano Niguarda, Milan, ITA

**Keywords:** chronic rhinosinusitis, functional endoscopic sinus surgery, inferior turbinoplasty, olfaction, quality of life

## Abstract

Functional endoscopic sinus surgery (FESS) is widely used to treat chronic rhinosinusitis (CRS) and related sinonasal diseases, often performed alongside inferior turbinoplasty to improve symptoms. However, the added value of concurrent turbinoplasty remains unclear. This systematic review evaluates outcomes of FESS with and without inferior turbinoplasty.

A systematic search of MEDLINE, Embase, and Cochrane databases was conducted following the Preferred Reporting Items for Systematic Reviews and Meta-Analyses (PRISMA) guidelines. After screening, five studies comprising 4,619 patients met the inclusion criteria.

Four studies assessed quality of life (QoL), all demonstrating postoperative improvement with both FESS alone and FESS plus inferior turbinoplasty. Only one study showed a significant QoL benefit with turbinoplasty; the others found no meaningful difference. One study evaluated olfaction and reported no significant impact from additional turbinoplasty. Revision surgery rates were not reported in any study. A paediatric study found no increased 30-day readmission risk associated with turbinoplasty.

Concurrent inferior turbinoplasty with FESS appears safe and may offer benefit in select patient subgroups, such as those with nasal polyps. However, routine addition does not consistently improve overall QoL or olfactory outcomes. Further randomised controlled trials are required to clarify its clinical value.

## Introduction and background

Inferior turbinate hypertrophy is a common finding in patients with sinonasal disease, contributing significantly to nasal obstruction and impaired airflow [1]. It most frequently occurs secondary to allergic or vasomotor rhinitis and compensatory hypertrophy from septal deviation [2,3]. Chronic inflammatory conditions cause collagen deposition beneath the basement membrane, leading to mucous gland hyperplasia, stromal oedema, and persistent mucosal swelling [4].

The inferior turbinates play an essential physiological role in regulating nasal airflow, humidification, and filtration, increasing surface area to optimise pulmonary gas exchange [5]. When enlarged, whether from mucosal or bony hypertrophy, they can substantially narrow the nasal airway, often producing greater obstruction than septal deviation, tonsillar hypertrophy, or nasal polyps [6,7]. Epidemiological studies suggest that inferior turbinate hypertrophy is present in up to one-third of patients undergoing evaluation for chronic nasal obstruction, underscoring its clinical relevance.

This functional importance has led some surgeons to combine functional endoscopic sinus surgery (FESS) with inferior turbinoplasty (IT) to optimise nasal airflow and postoperative outcomes. Turbinoplasty is an umbrella term encompassing several techniques designed to reduce turbinate volume while maintaining function [8]. Various methods include submucous diathermy (SMD), radiofrequency ablation, outfracturing, and turbinectomy (total, subtotal, or partial) [9]. These procedures can broadly be divided into mucosal-sparing approaches (SMD, radiofrequency ablation, and outfracture) and non-mucosal-sparing resections, such as partial or total turbinectomy. Mucosal-sparing procedures aim to preserve normal humidification and mucociliary function, whereas more extensive resections may increase the risk of postoperative crusting, dryness, and empty nose symptoms.

However, the role of concurrent IT during FESS remains debated within the otolaryngology community, particularly regarding its impact on olfactory function, revision surgery rates, and patient-reported quality of life (QoL) [6]. Long-term surgical success, often measured by the need for revision procedures, is another important but incompletely understood consideration [10]. Despite its frequent use, there remains no consensus on the added clinical value or optimal technique of turbinoplasty performed alongside FESS [11]. Given the heterogeneity of existing studies, such as spanning technique, population, and outcome measures, a systematic review is warranted to clarify the true effect of concurrent IT in endoscopic sinus surgery.

## Review

Materials and methods

The systematic review was developed and performed in accordance with the Preferred Reporting Items for Systematic Reviews and Meta-Analyses (PRISMA) 2020 statement to ensure methodological rigor and transparency while enabling reproducibility and reducing bias [12].

Search Strategy

A comprehensive literature search was conducted across MEDLINE, Embase, and Cochrane to identify relevant studies in March 2025. The review was registered on the International Prospective Register of Systematic Reviews (PROSPERO) (ID: CRD420251006186). The search was conducted using the Ovid platform and included a combination of keywords and subject headings targeting terms related to endoscopic sinus surgery and turbinate interventions. For endoscopic sinus surgery, the following terms were used in the title and abstract fields: endoscopic sinus surgery.ab,ti., FESS.ab,ti., ESS.ab,ti., sinus endoscopy.ab,ti., sinus surgery.ab,ti., and "nasal endoscop surgery".ab,ti.. These were combined using the OR operator. To capture relevant studies involving turbinate procedures, the following terms were also searched in the title and abstract fields: Turbinoplasty.ab,ti., turbinectomy.ab,ti., turbinate surgery.ab,ti., turbinate reduction.ab,ti., turbinate outfracture.ab,ti., "submuco diathermy".ab,ti., SMD.ab,ti., turbinate excision.ab,ti., turbinotomy.ab,ti., and (turbinate adj2 resection).ab,ti.. These terms were also combined with the OR operator. The two sets were then combined using the AND operator to ensure that all included studies addressed both endoscopic sinus surgery and turbinate-related interventions. This strategy was designed to maximise sensitivity while maintaining specificity for studies relevant to the review question. Additionally, the reference lists of included studies were manually screened for further eligible articles and to exclude grey literature.

Study Selection Process

The study selection process followed PRISMA guidelines. All records retrieved from database searches were imported into Covidence for deduplication. Two independent reviewers (SA and AM) screened the titles and abstracts, followed by the full-text screening of potentially eligible studies against a predefined inclusion and exclusion criteria for suitability. Disagreements were resolved through discussion or consultation with a third reviewer.

Inclusion and Exclusion Criteria

Studies were deemed eligible if they met the following criteria: included a comparison group of FESS+/-inferior turbinectomy, studies in the English language, and no age restriction. Exclusion criteria included the following: case reports, letters to the editor, conference abstracts, studies lacking relevant outcome measures, studies reporting on the superior or middle turbinates, and qualitative studies without comparison groups.

Data Extraction

Key study characteristics, including study design, population, outcomes, and year of publication, were extracted using a standardised data extraction form formulated on Microsoft Word (Microsoft Corporation, Redmond, Washington, United States). The form was piloted on two studies and revised accordingly.

Two independent reviewers extracted data from each eligible study, resolving discrepancies through discussion with a third and fourth reviewer. Final data categories included QoL symptom scoring and olfactory assessment.

Outcomes

The following predefined outcomes were evaluated: QoL, olfaction function, and the need for revision surgery. The assessed outcomes are detailed below.

Risk of Bias

Each study was evaluated for biases related to selection, performance, attrition, and reporting. Two independent reviewers conducted the risk of bias assessment, and discrepancies were resolved through discussion.

Results

Study Selection

**Figure 1:**
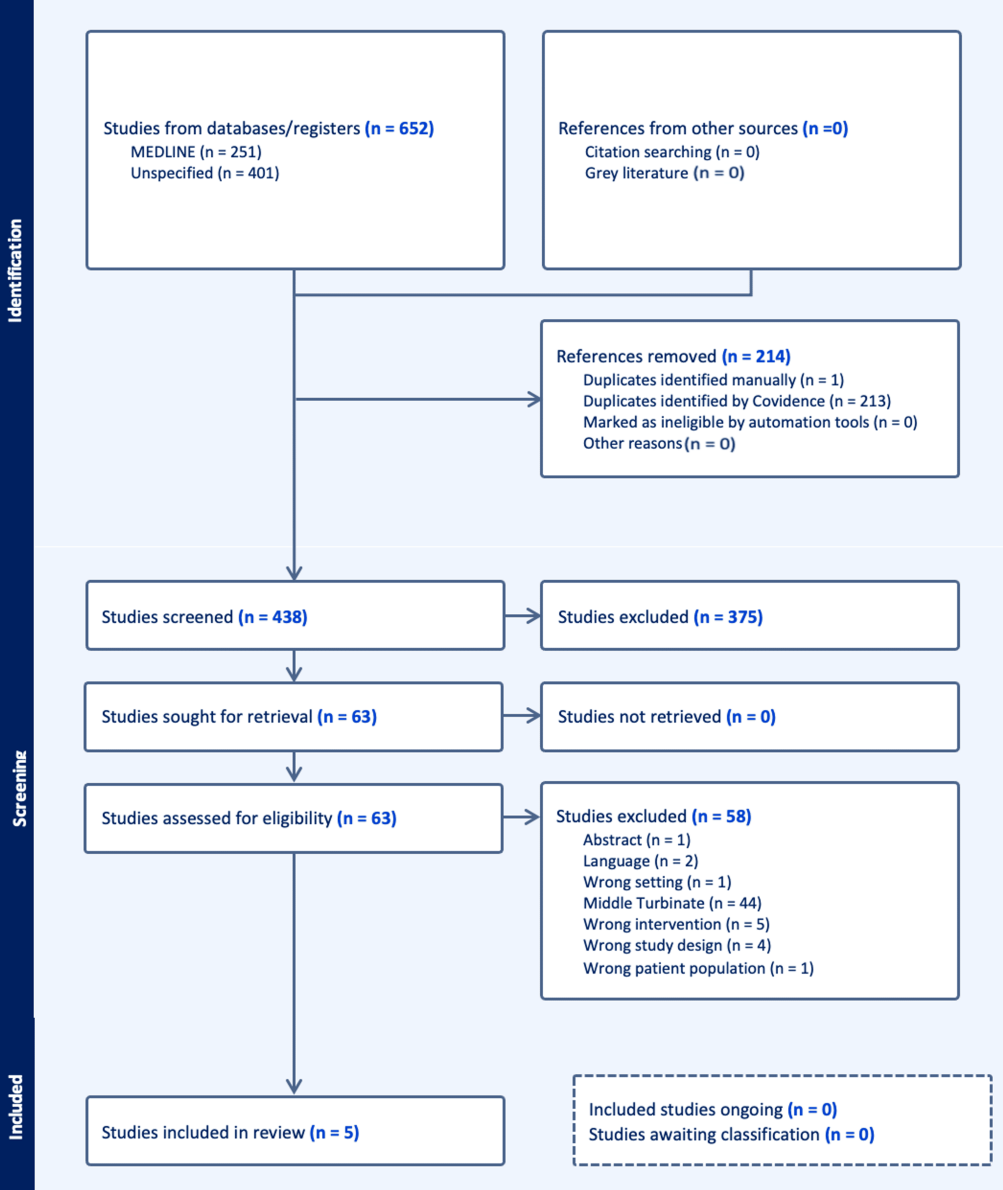
PRISMA flow diagram The PRISMA flow diagram outlines the process of study identification, screening, eligibility assessment, and inclusion in the systematic review. The diagram depicts the number of records identified through database searching and other sources, the number of duplicates removed, records screened, full-text articles assessed for eligibility, and studies included in the final qualitative and/or quantitative synthesis. PRISMA: Preferred Reporting Items for Systematic Reviews and Meta-Analyses

A total of 652 studies were identified through database searches (Embase: X; Scopus: Y). After removing 214 duplicates (213 via Covidence and one manually), 438 unique articles remained for screening. Title and abstract screening excluded 375 studies that did not meet the predefined eligibility criteria. This yielded 63 full-text articles for detailed evaluation. Of these, 58 were excluded, primarily due to the absence of a direct comparison between FESS with and without turbinoplasty. Five studies fulfilled the eligibility criteria and were included in this systematic review. The PRISMA flow diagram (Figure 1) outlines the study selection process.

Study Characteristics

The five included studies are summarised in Table 1 [5,13-16]. They consisted of a randomised controlled trial and prospective or retrospective observational cohort studies published between 2012 and 2023. Sample sizes ranged from 80 to 2,205 patients, collectively evaluating over 4,000 participants undergoing FESS with or without turbinoplasty.

**Table 1:** Study characteristics The table presents key details of each study, including author(s) and year of publication, study design, sample size, surgical intervention, outcome measures, and follow-up period. Study designs vary across the included studies, and sample sizes range from 80 to 2986 participants. Outcome measures differ between studies, reflecting the heterogeneity of the included research. RCT: randomised controlled trial; CRS: chronic rhinosinusitis; FESS: functional endoscopic sinus surgery; SMD: steroid-eluting sinus stent or spacer; NES: nasal endoscopy score; SNOT-22: 22-Item Sinonasal Outcome Test; BITR: balloon intervention of the frontal recess; EQ-5D: EuroQol 5-Dimension Questionnaire; CSS: Chronic Sinusitis Survey; RSDI: Rhinosinusitis Disability Index; SF-36: 36-Item Short-Form Health Survey; SIT: Smell Identification Test; TR: turbinate reduction

Author	Country	Year	Study design	Population	Sample size	Intervention	Control	Outcome measured	Follow-up period
Manimaran et al. [5]	India	2023	RCT	Adults CRS	80	FESS+SMD	FESS	NES, modified SNOT, modified Lund-Kennedy endoscopic scoring	1, 2, 3 months
Scangas et al. [13]	US	2019	Cohort study	Adults CRS	901	FESS+BITR	FESS	EQ-5D, SNOT-22, CSS	3, 12, 24, 36, 48 months
Soudry et al. [14]	US	2019	Cohort study	Adults CRS	571	FESS+BITR	FESS	SNOT-22, RSDI, CSS, SF-36, Lund-Mackay CT score, SIT score	6, 12, 18 months
McKeon et al. [15]	US	2019	Retrospective cohort study	Paediatric	2986	FESS+turbinoplasty	FESS	Readmission no. (%)	30 days
Murthy and Banerjee [16]	UK	2013	Cohort study	>16 years CRS	93	FESS+TR	FESS	Symptom score	4, 12 months

Two studies [13,14] included adult patients with chronic rhinosinusitis (CRS), while one study [16] also included patients with recurrent acute sinusitis and nasal polyposis and another with inferior turbinate hypertrophy and CRS [5]. The fifth study [15] focused on paediatric patients (<18 years) who underwent endoscopic sinus surgery for CRS and were readmitted within 30 days postoperatively. Follow-up periods ranged from one month to four years, allowing for the assessment of both short- and long-term outcomes.

Regarding surgical techniques, two [5,14] studies utilised SMD, a mucosal-sparing approach for inferior turbinate reduction. One study [13] left the choice of turbinoplasty to the surgeon's discretion, while two [15,16] did not specify the technique. Only one study randomised patients to either FESS with or without turbinoplasty, while in two studies, the decision to perform turbinoplasty was based on the surgeon's evaluation of patient symptoms, endoscopic findings, and radiologic results. Two studies did not report the selection criteria.

Outcomes and Findings

None of the included studies reported on the need for revision surgery. Four studies evaluated QoL using various patient-reported outcome measures. Two used validated instruments, namely, the 22-Item Sinonasal Outcome Test (SNOT-22) and the Chronic Sinusitis Survey (CSS), while another employed a modified seven-item version of SNOT, and one utilised a 10-point visual analogue scale (VAS) (Table 2). Other QoL scoring systems used also included the Rhinosinusitis Disability Index (RSDI), a standardised 30-item survey, EuroQol 5-Dimension Questionnaire (EQ-5D), Short-Form 6-Dimension Health Utility Survey (SF-6D HUS), and 36-Item Short-Form Health Survey (SF-36). Olfactory function was evaluated in one study using the Brief Smell Identification Test (B-SIT) and Smell Identification Test (SIT), a 12-item test and a 40-item test, respectively. In addition, the included studies reported other outcomes, including the Lund-Kennedy endoscopic score, the number of complications, and 30-day readmission rates. The results are summarised in Table 2 [5,13-16] and Table 3 [5,13-16].

**Table 2:** Quality of life outcomes Quality of life outcomes include the SNOT-22, CSS, NES, RSDI, EQ-5D, symptom score (a visual analogue scale-based composite of sinonasal symptom severity as described by Murthy et al. [16]), SF-6D HUS, and SF-36 general health. SNOT-22: 22-Item Sinonasal Outcome Test; CSS: Chronic Sinusitis Survey; NES: nasal endoscopy score; RSDI: Rhinosinusitis Disability Index; EQ-5D: EuroQol 5-Dimension Questionnaire; SF-6D HUS: Short-Form 6-Dimension Health Utility Survey; SF-36: 36-Item Short-Form Health Survey; FESS: functional endoscopic sinus surgery; BITR: balloon intervention of the frontal recess

Study	SNOT-22 (+ modified)		CSS		NES		RSDI total		EQ-5D		Symptom score		SF-6D HUS		SF-36 general health	
	FESS	FESS+BITR	FESS	FESS+BITR	FESS	FESS+BITR	FESS	FESS+BITR	FESS	FESS+BITR	FESS	FESS+BITR	FESS	FESS+BITR	FESS	FESS+BITR
Manimaran et al. [5]	9.00±3.08	6.00±1.81	-	-	4.05±1.66	2.38±0.89	-	-	-	-	-	-		-	-	-
Scangas et al. [13]	-21.0	-28.2	23.41	34.29	-	-	-	-	0.077	0.083	-	-	-	-	-	-
Soudry et al. [14]	-24.3	-27.8	21.2	35.6	-	-	-19.7	-25.3	-	-	-	-	0.09	0.08	4.5	10.6
McKeon et al. [15]	-	-	-	-	-	-	-	-	-	-	-	-	-	-	-	-
Murthy and Banerjee [16]	-	-	-	-	-	-	-	-	-	-	0.12	3.9 (-3.9 to 11.6)	-	-	-	-

**Table 3:** Clinical outcomes Clinical outcomes included Lund and Kennedy score, number of complications, SIT and B-SIT, and percentage of readmissions. Manimaran et al. [5] utilised a modified SNOT scoring system using only seven parameters and a modified Lund-Kennedy endoscopic scoring system using four parameters. Results were described as the mean±standard deviation (SD) at the three-month period. Results by Scangas et al. [13] and Soudry et al. [14] were recorded as a change in the mean outcome measure over time at the end of the study. Scangas et al. [13] noted five cases of epistaxis requiring admission following surgery, where one of those cases underwent BITR. McKeon et al. [15] recorded the number of readmissions in FESS with turbinectomy, which showed no significant difference compared to FESS. Murthy and Banerjee [16] assessed symptoms scores at 12 months using linear regression analysis. Results were noted as follows: Effect (95% CI). SIT: Smell Identification Test; B-SIT: Brief Smell Identification Test; FESS: functional endoscopic sinus surgery; BITR: balloon intervention of the frontal recess; SNOT: Sinonasal Outcome Test

Study	Lund and Kennedy (+ modified)		Complications		SIT		B-SIT		Readmission %	
	FESS	FESS+BITR	FESS	FESS+BITR	FESS	FESS+BITR	FESS	FESS+BITR	Total FESS	FESS+BITR
Manimaran et al. [5]	3.35±1.46	2.50±0.84	-	-	-	-	-	-	-	-
Scangas et al. [13]	-	-	4/788	1/113	-	-	-	-	-	-
Soudry et al. [14]	-3.1	-1.9	-	-	2.2	0	0.1	0.7	-	-
McKeon et al. [15]	-	-	-	-	-	-	-	-	4%	3.9%
Murthy and Banerjee [16]	-	-	-	-	-	-	-	-	-	-

QoL

All four studies [5,13,14,16] evaluating QoL demonstrated significant postoperative improvement in both FESS-only and FESS+BITR (bilateral inferior turbinate reduction) groups. However, only one of these studies [5] demonstrated that the FESS+BITR group had superior QoL outcomes compared with the group without BITR.

One study [14] again found significant improvement in CSS, SNOT-22, and RSDI scores when either a FESS was performed with or without BITR. However, in the BITR group, the improvement in CSS, SNOT-22, and RSDI was not clinically meaningful as it did not exceed the minimum clinically important difference (MCID). Also, when examining specifically the nasal blockage/congestion item of SNOT-22, although nasal blockage symptoms improved more in the BITR group, the difference was not statistically significant. In a subgroup analysis, patients with chronic rhinosinusitis with nasal polyps (CRSwNP) showed significantly greater improvement in nasal obstruction with BITR (-3.67±1.86 vs. -2.32±1.78; p=0.006). However, this difference was not significant in the chronic rhinosinusitis without nasal polyps (CRSsNP) subgroup (2.24±1.60 vs. 1.6±1.50; p=0.076).

Another study [13] reported significant improvements in SNOT-22, CSS, and EQ-5D scores over a four-year follow-up in both groups, with no significant difference between FESS alone and FESS+BITR. Multivariate analysis confirmed that BITR was not an independent predictor of superior QoL improvement. The third study [16], comparing FESS alone versus FESS+SMD, found no statistically significant difference in symptom scores at 12 months (mean improvement: 3.9 points greater with FESS+SMD; p=0.321).

One study [5] reported that FESS+BITR resulted in significantly greater improvement in NES, modified SNOT, and modified Lund-Kennedy scores at the three-month follow-up compared to FESS alone. However, the short follow-up period (three months) limited the generalisability of these findings.

Olfaction

The study assessing olfaction using the SIT found that BITR was not independently associated with significant improvement in full SIT scores, as defined by an increase of at least one MCID value [14].

Paediatric Readmission Rates

The final study, which examined 30-day readmission rates in paediatric patients, found no increased risk associated with adjunctive turbinoplasty [15]. Among the 426 patients who underwent concurrent turbinectomy, none required readmission.

Quality Assessment

**Figure 2:**
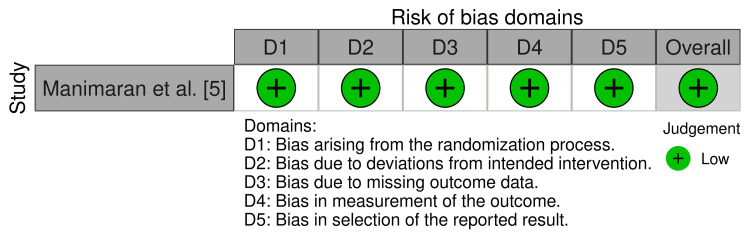
Quality assessment using the Cochrane Risk of Bias 2.0 (RoB 2) tool Quality assessment using the Cochrane Risk of Bias 2.0 (RoB 2) tool for randomised controlled trials demonstrated an overall low risk of bias.

The randomised controlled trial included in this study was assessed using the Cochrane Risk of Bias 2.0 (RoB 2) tool, while the cohort studies included were assessed using the Newcastle-Ottawa Scale (NOS) (Table 4 [13-16] and Figure 2) [17,18]. Overall, the studies included were of high quality with low risk of bias.

**Table 4:** Quality assessment and risk of bias The Newcastle-Ottawa Scale (NOS) was used to assess the quality of cohort studies included in this systematic review. Scangas et al. [13] demonstrated strong selection and comparability components and robust outcome measurement. However, high dropout rates especially in later years could introduce potential attrition bias (down to 31% by year 4). Soudry et al. [14] also produced a high-quality study scoring 8/9 due to moderate loss to follow-up (~35%). McKeon et al. [15] scored 8/9 stars as it lacked a comparison to a non-surgical control group. Murthy and Banerjee [16] scored 9/7 for lack of a non-exposed cohort and limited information on follow-up completeness.

Study	Representativeness of the exposed cohort	Selection of the non-exposed cohort	Ascertainment of exposure	Outcome not present at the start	Comparability of cohorts (design or analysis)	Assessment of outcome	Was follow-up long enough for outcomes to occur?	Adequacy of follow-up of cohorts	Total
Scangas et al. [13]	☆	☆	☆	☆	☆☆	☆	☆	x	8/9
Soudry et al. [14]	☆	☆	☆	☆	☆☆	☆	☆	x	8/9
McKeon et al. [15]	☆	x	☆	☆	☆☆	☆	☆	☆	8/9
Murthy and Banerjee [16]	☆	x	☆	☆	☆☆	☆	☆	x	7/9

Discussion

Patient-reported outcome measures, such as the validated SNOT-22, are increasingly recognised as a key measure of surgical success. This evaluates symptom burden, QoL, and postoperative well-being from the patient's perspective in the form of a validated questionnaire. Although FESS alone has demonstrated significant improvements in SNOT-22 scores, the additive effect of turbinoplasty on these outcomes remains controversial [19]. Some studies report enhanced symptom relief when turbinoplasty is performed alongside FESS, whereas others suggest that the effect is minimal or transient [13].

This systematic review found that while both FESS alone and FESS with IT result in significant postoperative improvement, the added benefit of IT remains debatable. Notable improvements in QoL were seen in all the studies, regardless of the addition of IT, although only one reported a significant improvement in QoL outcomes with the inclusion of IT [5]. This study, however, has the shortest follow-up period of three months, limiting its reliability. The other studies found no statistically significant difference, with the combination of IT and FESS compared to FESS alone. Soudry et al. [14] found a significant difference between pre- and postoperative outcomes for both FESS and FESS with ITR. However, statistical tests were not employed to assess the significance of the difference between both interventions, which gives us limited information about the effectiveness of including ITR.

Further subgroup analyses in one of the papers suggested CRSwNP may derive greater benefit from IT surgery. However, this effect was not observed in those without nasal polyps, demonstrating that there might be disease phenotype/endotype-specific benefits of IT despite no overall benefit. This finding warrants further investigation to better clarify which subsets of CRS patients would benefit most from the addition of IT to FESS.

Olfactory function is a critical but unfortunately often overlooked aspect of turbinate surgery. The nasal turbinates play a crucial role in airflow dynamics, in directing odorants toward the olfactory cleft. Thus, surgical modification could theoretically either improve or impair the sense of smell, with some studies suggesting improvement of olfactory function by improving airflow dynamics, while others indicate concerns about disruption to the olfactory pathway and causing empty nose syndrome [10]. Empty nose syndrome arises when excessive resection or loss of turbinate mucosa leads to abnormal nasal aerodynamics, dryness, crusting, and a paradoxical sensation of nasal obstruction despite a patent airway. This condition can markedly affect QoL and underscores the importance of mucosal preservation in turbinate surgery as postoperative inflammation, crusting, and mucosal healing may influence olfactory recovery [3]. Another study looking at the olfactory function in patients undergoing septoplasty with partial inferior turbinectomy found that although most patients experienced postoperative olfactory improvement, approximately 20% exhibited a moderate decrease in olfactory function, suggesting that IT can adversely affect the sense of smell in a subset of individuals [20].

Furthermore, the study evaluating paediatric readmission rates found no increased risk associated with additional IT at 30 days [15]. This suggests that, at least in paediatric populations, the addition of inferior turbinectomy does not increase morbidity in the short term postoperatively. This addresses a common concern among surgeons regarding potential complications and the safety and feasibility in this population, although larger paediatric studies are needed to confirm this finding.

The results of this review are congruent with existing literature, which reports mixed outcomes. Some studies highlight that combining IT with FESS can cause an improvement in nasal congestion and airflow. For example, one study demonstrated a significant postoperative improvement in mucociliary clearance when assessing mucociliary function in patients undergoing nasal surgeries, including FESS+IT [21]. On the other hand, other studies have shown a negative outcome. For example, research has suggested an inferior turbinectomy can predispose patients to sinus infections, possibly because of alterations in nasal physiology and mucociliary clearance, causing patients to develop chronic sinusitis postoperatively [22]. Furthermore, in a randomised controlled trial looking at QoL outcomes between patients with endoscopic partial inferior turbinectomy (EPIT) combined with primary rhinoseptoplasty and rhinoseptoplasty on their own, no significant difference was found in the two groups [23]. This suggested the addition of EPIT increases surgical time and potential general anaesthesia-associated morbidity without any significant improvement in postoperative outcomes.

For instance, a 20% overall complication rate was found in a retrospective study evaluating early complications in patients undergoing inferior turbinectomy [24]. Nine percent of cases also had significant haemorrhage, and other complications included adhesions, crusting, infection, and septal perforation. Another study on patients undergoing the surgery reported complications such as synechiae (15%), atrophic rhinitis (15%), persistent obstruction (12%), and abnormal nasal sensation (9%) [25]. These complications demonstrate the potential complications of the surgery and how it can negate the intended benefits. A retrospective study on the surgical outcomes of inferior turbinectomy in mucormycosis patients highlighted a high recurrence of 21%, demonstrating the problem of relying solely on the surgery without tackling systemic factors such as glycaemic control, and emphasises that the routine addition of inferior turbinectomy should be reserved for specific patient profiles rather than applied universally [26].

Clinical Implications

The findings of this review suggest that while concurrent IT during FESS is safe and may improve nasal airflow and symptom relief in select patients, particularly those with CRSwNP, its routine use cannot be universally recommended. Careful patient selection and preference for mucosal-sparing techniques are advised to minimise postoperative complications such as crusting or empty nose syndrome. Surgeons should individualise the decision to perform IT based on objective turbinate hypertrophy, symptom burden, and intraoperative assessment, rather than as a standard adjunct to FESS.

Strengths and Limitations

This systematic literature review supplies a thorough integration of the current evidence on FESS with and without inferior turbinate surgery. The inclusion of both adult and paediatric populations, with the inclusion of multiple outcomes, including QoL and olfaction, enhances the generalisability of the results.

However, several limitations must be acknowledged. Firstly, there is heterogeneity between the papers. This includes heterogeneity in the surgical technique (e.g., SMD, radiofrequency ablation), population, outcomes (as well as the tools used to assess these outcomes), and follow-up period, which overall limits the ability to draw definitive conclusions. In particular, the included studies varied in their definitions and execution of IT, ranging from mucosal-sparing procedures such as SMD and radiofrequency ablation to more extensive, non-mucosal-sparing resections such as partial turbinectomy. Because most studies did not clearly describe or standardise their chosen technique, a meta-conclusion across all forms of IT must be interpreted with caution. Future research should therefore stratify results by technique type, such as mucosal-sparing versus non-mucosal-sparing methods, to better clarify their relative safety, functional outcomes, and patient-reported benefits.

Secondly, only one study incorporated randomisation, while the remaining studies were observational cohorts, adding risk of selection bias. Thirdly, some studies had a short follow-up period of three months, which could underestimate long-term benefits. Finally, there was exclusion of non-English studies and grey literature in this literature review, which may have introduced publication bias, potentially skewing the results toward studies with significant findings.

## Conclusions

The routine addition of IT surgery to FESS may not be universally beneficial. While it showed surgery did offer additional symptom relief in select patient subgroups, such as those with CRSwNP, its overall effect on QoL and olfactory outcomes appears limited; thus, the results should be interpreted with caution. There is evidence for a patient selection criterion to be applied for this surgery, potentially limiting the use of IT surgery to patients with significant inferior turbinate hypertrophy or with nasal polyps.
